# Impact of genetic polymorphisms at the promoter area of IL-10 gene on tacrolimus level in Jordanian renal transplantation recipients

**DOI:** 10.5937/jomb0-33343

**Published:** 2022-07-29

**Authors:** Bara'ah Khaleel, Al-Motassem Yousef, Mazhar Salim Al-Zoubi, Muhammad Al-Ulemat, Ahmad A. Masadeh, Ali Abuhaliema, Khalid M. Al-Batayneh, Bahaa Al-Trad

**Affiliations:** 1 Yarmouk University, Faculty of Science, Department of Biological Sciences, Irbid, Jordan; 2 The University of Jordan, School of Pharmacy, Department of Biopharmaceutics and Clinical Pharmacy, Amman, Jordan; 3 Yarmouk University, Faculty of Medicine, Department of Basic Medical Sciences, Irbid, Jordan; 4 Royal Medical Services, Pharmacy Department, Amman, Jordan

**Keywords:** IL-10 genetic polymorphism, kidney transplantation, pharmacokinetics, tacrolimus, IL-10 genetski polimorfizam, transplantacija bubrega, farmakokinetika, takrolimus

## Abstract

**Background:**

Tacrolimus is a widely used immunosuppressant that prevents solid organ transplant rejection. The pharmacokinetics of Tacrolimus show considerable varia - bility. Interleukin-10 (IL-10), in the host's immune response after transplantation, contributes to the variable CYP3Adependent drug disposition of Tacrolimus. In the current study, we aim to evaluate the impact of single nucleotide polymorphisms (SNP) in the promoter region of IL-10 on Tacrolimus dose requirements and the Dose Adjusted Concentration (DAC) of Tacrolimus among kidney transplantation recipients.

**Methods:**

Blood levels of Tacrolimus were measured using Microparticle Enzyme Immunoassay (MEIA) for six months post-transplantation. Genotyping analysis was utilized using specific Polymerase Chain Reaction (PCR) followed by sequencing methods for 98 Jordanian kidney transplant recipients.

**Results:**

Genotyping frequencies of IL-10 (-592) were (CC/CA/AA: 38, 46.7, 15.2%); IL-10 (-819) were (CC/CT/TT: 40.4, 44.1, 15.1%); and IL-10 (-1082) were (AA/AG/GG: 42.6, 44.7, 12.8%). The impact of IL-10 (-1082) on Tacrolimus DAC was gender dependent. Men carrying at least one A allele had significantly lower DAC than men carrying GG genotyping only in the first month post-transplantation 88.2±32.1 vs. 117.5±22.5 ng/mL per mg/kg/day, p=0.04 .

**Conclusions:**

Our current study showed that the interaction between gender and IL-10 -1082 affects Tacrolimus DAC in Jordanian kidney transplant recipients during the first month post-transplantation.

## Abstract (sr)

**Uvod:**

Takrolimus je široko korišćen imunosupresiv koji sprečava odbacivanje presađenog čvrstog organa. Farmako-kinetika takrolimusa pokazuje značajnu varijabilnost interleukin-10 (IL-10), u imunološkom odgovoru domaćina nakon transplantacije, doprinosi promenljivoj dispoziciji leka zavisne od CIP3A kod takrolimusa. U ovoj studiji, cilj nam je da procenimo uticaj polimorfizama pojedinačnih nukleotida (SNP) u promotorskom regionu IL-10 na zahteve za dozom takrolimusa i koncentraciju prilagođenu dozi (DAC) takrolimusa među primaocima transplantacije bubrega.

**Metode:**

Nivoi takrolimusa u krvi su mereni korišćenjem imunoeseja mikročestica enzima (MEIA) tokom šest meseci nakon transplantacije. Analiza genotipizacije je izvedena korišćenjem specifične lančane reakcije polimeraze (PCR) praćene metodama sekvenciranja za 98 jordanskih primalaca transplantiranih bubrega.

**Rezultati:**

Učestalosti genotipizacije IL-10 (-592) su (CC/CA/AA: 38, 46,7, 15,2%); IL-10 (-819) su (CC/CT/TT: 40,4, 44,1, 15,1%); i IL-10 (-1082) su (AA/AG/GG: 42,6, 44,7, 12,8%). Uticaj IL-10 (-1082) na takrolimus DAC zavisio je od pola. Muškarci koji su nosili najmanje jedan alel A imali su značajno niži DAC od muškaraca koji su nosili genotipizaciju GG samo u prvom mesecu nakon trans plantacije 88,2±32,1 naspram 117,5±22,5 ng/mL po mg/kg/dan, p=0,04 .

**Zaključak:**

Naša trenutna studija je pokazala da interakcija između pola i IL-10-1082 utiče na takrolimus DAC kod jordanskih primalaca transplantacije bubrega tokom prvog meseca nakon transplantacije.

## Introduction

End-stage renal disease (ESRD) is the final stage of kidney failure, characterized by a decreased glomerular filtration rate and increased urinary albumin excretion [1]. Kidney transplantation is considered the most effective treatment of End-stage renal disease (ESRD) when compared to dialysis [2] [3]. In 1954, Murray, and Merrill [4] performed the first successful kidney transplant operation; it was made possible because the donor and recipient were monozygotic identical twins. The first kidney transplantation in the Arab world was performed in Jordan in 1972, the kidney was obtained from a deceased donor [5].

Transplantation patients during their post-operative phase run a great risk of developing major life-threatening complications which include: (1) cardiovascular diseases most likely caused by calcification of vessels and left ventricular hypertrophy associated originally with ESRD; (2) delayed graft function which is defined as the use of dialysis due to poor kidney function in the first week of graft life; (3) infection due to the high level of immunosuppressants given to the patient, but the improved use of antimicrobials and antimicrobial regimens has decreased infection severity; (4) graft rejection [6] [7] [8] [9].

To solve these complications, immunosuppressants are essential for successful organ transplantation as they suppress rejection and inhibit the autoimmune process, however, they also lead to undesired consequences such as immunodeficiency, infection or malignancy, and non-immune toxicity [10]. Tacrolimus is a fermentation product of *Streptomyces *and belongs to the family of calcineurin inhibitors. It is a widely used immunosuppressive drug for preventing solid-organ transplant rejection [11]. But its usage is complicated due to its narrow therapeutic index and considerable inter-and intra-individual pharmacokinetic variability [12].

Many single-nucleotide polymorphisms (SNPs) have been studied concerning the pharmacokinetics of Tacrolimus, especially CYP3A4, CYP3A5, and ABCB-1; as their alleles have been involved in the metabolism of calcineurin inhibitors showing promising prospects in Tacrolimus dose individualization [13].

The IL-10 promoter area is highly polymorphic; many studies have been conducted showing variations in IL-10 expression linked to promoter area polymorphisms. Five SNPs tagging the promoter area of IL-10 have been widely studied and they are (-3575), (- 2763), (-1082), (-819), and (-592) [14].

IL-10 production level showed that the GG genotype -1082 is higher versus (AA and AG) genotypes, independently of the polymorphisms at positions -819 and -592, and also associated with higher serum concentration [15] [16] [17]. Furthermore, *in vivo* study showed that higher IL-10 decreases CYP3A activity, which is involved in the metabolism of tacrolimus among renal transplantation recipients [18] [13].

Our current study aimed to investigate the association between the dose required to reach the target level of Tacrolimus and genetic variations in renal transplantation recipients through the study of IL-10 (-592, rs1800872, A/C); IL-10 (–819, rs1800871, T/C) and IL-10 (–1082, rs1800896, A/G). The SNPs were selected due to the reported relationship with IL-10 production and level [15] [19].

## Materials and methods

### Patients and ethical approval

Ninety-eight adult renal transplant recipients, who had received a renal graft between 2009 and 2011 from Jordanian Royal Medical Services, were included in the study. The inclusion criteria were patients with a newly transplanted kidney and who were on a Tacrolimus-based immunosuppressive maintenance therapy starting immediately following transplantation. Tacrolimus was given in two equally divided doses. All patients treated with Tacrolimus used the capsule formulation Prograf^®^ (Fujisawa, Munich, Germany). Patients who received medications known to interact with Tacrolimus were excluded from the study.

This study was approved by local Research Ethics Committees of Jordanian Royal Medical Services (IRB: TF1/3/ethics obtained on June 27th, 2016); and has been performed following the ethical standards laid down in the 2000 Declaration of Helsinki as well as the Declaration of Istanbul 2008. Written informed consent was obtained from all participants. Details that might disclose the identity of the subjects in the study were omitted.

### Tacrolimus blood level measurements

Blood samples were collected before the administration of the morning dose of Tacrolimus for the determination of trough blood concentrations of the drug. The trough concentration was measured in whole blood by IMx Tacrolimus II assay which utilizes MEIA in the Abbott IMx system (Tacrolimus II; Abbott Laboratories, IL. USA). This measurement was performed in the laboratory of Jordanian Royal Medical Services, and the dose-adjusted concentration was calculated by dividing the pre-dose concentration by the corresponding 24-hour dose in milligram Tacrolimus per kilogram body weight.

### Genomic DNA isolation and genotype analysis

Genomic DNA was isolated from 300 μL EDTA-treated whole blood using a Commercial kit (Wizard genomic DNA purification kit, Promega, WI, USA). The procedure was carried according to the kit manufacturer's recommendation.

Genotyping analysis for detection of 3 SNPs of IL-10s was performed for all patients by using specific PCR primers. Table 1 describes primers used and PCR conditions. PCR was performed in a total volume of 25 μL using 100 ng of genomic DNA with 1.5 μL of 10 μmol/L of each primer and 12.5 μL of 2X KAPA2G Fast ReadyMix PCR Kit (Kappa Biosystems, USA). PCR amplifications were performed in PTC-100 Peltier Thermal Cycler (MJ Research, MA, USA).

**Table 1:** Primers, PCR conditions of genotyping analysis for *IL-10 -1082A/G*, *IL-10 -592A/C* and *IL-10 -819T/C*

Allele	Positiona	Primers	PCR conditions
IL-10 -1082A/G	rs1800896	Forward primer 5’ GGCTTCCTACAGTACAGGCG 3’<br> Reverse Primer 5’ GGTAGAGCAACACTCCTCGC 3’	Denaturing 95 °C for 1 min<br>Annealing 60 °C for 1 min<br>Extension 72 °C for 1 min<br>35 cycles<br>Size of PCR product<br>447 bp and 783 bp
IL-10 –592A/CIL-10–819T/C	rs1800872<br> rs1800871	Forward primer 5’GATGAATACCCAAGACTTCTCCT3’<br>Reverse Primer 5’CCTTCCCCAGGTAGAGCAACAC3’	

PCR reaction products were sequenced using Big Dye Terminator version 3.1 kit (Applied Biosystems, Waltham, MA, USA). Samples were run on an ABI Prism Genetic Analyzer system 3130xl (Applied Biosystems, Waltham, MA, USA).

### Statistical analysis

Data were coded and entered into Statistical Packages for Social Sciences (SPSS version 20.0, 2012). Data were summarized as counts and percentages for categorical data and as means and standard deviation (SD) for continuous data. A data set was tested for normality of distribution using the Shapiro-Wilk test. Homogeneity of variance was assessed by Levene's test. Comparison between categorical data was conducted using the Fisher exact test or Chi-square test, when appropriate. Comparison between continuous data was performed utilizing independent t-test; ANOVA, Mann-Whitney or Kruskal Wallis, based on which was most appropriate. A *p*-value of 0.05 or less was considered statistically significant.

## Results

Ninety-eight kidney transplant recipients met our inclusion criteria. The age, weight and gender of donors and recipients are comparable. Demographic data of recipients and corresponding donors are summarized in Table 2. The most common cause of chronic kidney disease among our patients was hypertensive nephropathy (49%). Other identifiable causes of chronic kidney disease included glomerulonephritis (8.2%), chronic pyelonephritis (6.1%), diabetic nephropathy (4.1%), and polycystic kidney disease (4.1%). Ninety-seven percent of patients underwent the transplantation operation for the first time, with the majority of them receiving the graft from a living relative (93%). The medical history of kidney transplant recipients is summarized in Table 3.

**Table 2:** Demographic data of Jordanian kidney transplant recipients and corresponding donors Chi-Squared with one degree of freedom

Parameter		Donors	Recipients	p
Gender N<br>(%)	Male	53 (54.1%)	60 (61.2%)	0.38
	Female	45 (45.9%)	38 (38.8%)	
Age, years, mean (±SD)		34.1±8.9	35.6±9.6	0.26
Weight, kg, mean (±SD)		70.9±16.4	72.1±17.4	0.62

**Table 3:** Medical history data for Jordanian kidney transplant recipients N: number of recipients. SD: standard deviation

Parameter		N (98)
Causes of<br> chronic<br> kidney disease,<br>N (%)	Glomerulonephritis	8 (8.2%)
	Chronic pyelonephritis	6 (6.1%)
	Diabetic nephropathy	4 (4.1%)
	Hypertensive nephropathy	48 (49.0%)
	Polycystic kidney disease	4 (4.1%)
	Undetermined	8 (8.2%)
	Others	20 (20.4%)
Transplantation<br> events<br>N (%)	First	95 (96.9%)
	Second	3 (3.1%)
Type of donors<br>N (%)	Relative, living	91 (92.9%)
	Non relative, living	7 (7.1%)
Immunosuppressant use		
Prednisolone N (%)		95 (96.9%)
	Total daily dose<br>(mean±SD), mg	11.8±6
Azathioprine N (%)		6 (6.1%)
	Total daily dose<br>(mean±SD), mg	75 ± 27.4
Mycophenolate N (%)		89 (90.0%)
	Total daily dose<br>(mean±SD), mg	1393.3±491.2

### Genotypes and alleles frequencies

Among the 98 kidney transplant recipients, some cases were not genotyped due to unsuccessful PCR results. The allele frequencies of the 3 SNPs in all patients were in accordance with the Hardy-Weinberg Equilibrium equation (P>0.05). Genotype frequencies of patients are summarized in Table 4.

**Table 4:** Genotype frequencies of Jordanian kidney transplant recipients N: number of recipients. χ^2^: Chi-Squared with one degree of freedom

Genotype	N (%)		Allele frequency<br>%		X^2^	P
IL-10<br>(-592,rs1800872,C/A)	CC	35 (38)	Minor	61	0.01	0.99
	CA	43 (46.7)	Major	36		
	AA	14 (15.2)				
IL-10<br>(–819,rs1800871,C/T)	CC	38 (40.9)	Minor	37	0.1	0.96
	CT	41 (44.1)	Major	63		
	TT	14 (15.1)				
IL-10<br>(–1082,rs1800896,A/G)	AA	40 (42.6)	Minor	34	0.06	0.97
	AG	42 (44.7)	Major	66		
	GG	12 (12.8)				

### Effect of recipients genotypes on Tacrolimus pharmacokinetics parameters

**Figure 1:**
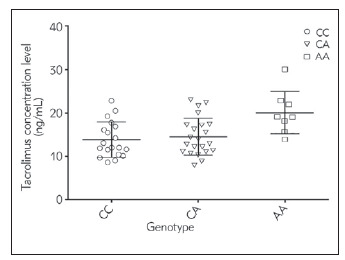
Effect of gender-genotype at IL-10 (-592, rs1800872, C/A) on Tacrolimus concentration level (ng/mL) of Jordanian kidney transplant recipients in the first month post transplantation Y-axis is Tacrolimus concentration level (ng/mL), X-axis is the genotypes at IL-10 -592. CC: Homozygous ancestral genotype, AA: Homozygous variant genotype and CA: Heterozygote variant genotype

Daily dose (mg/day), concentration level (ng/mL), weight-adjusted daily dose (mg/kg/day), body surface area (BSA) adjusted dose (mg/m^2^) and dose-adjusted concentration (DAC) (ng/mL per mg/kg/day) of Tacrolimus were compared among recipients with different allelic statuses of three SNPs of *IL-10*. All mentioned parameters did not differ significantly among *IL-10* (–819, rs1800871, T/C) and IL-10 (–1082, rs1800896, A/G) during the first six months post-transplantation as shown in Supplementary Tables (Table 1 and Table 3). However, we found that patients carrying AA at *IL-10* (-592, rs1800872, A/C) had significantly a higher tacrolimus concentration level than those patients carrying AC or CC genotypes in the first month, post-transplantation (AA: 20.1±4.95 ng/mL; AC: 14.58±4.4 ng/mL; and CC: 13.86±4.1 ng/mL, *p* = 0.01). This difference in Tacrolimus concentration level disappeared after the first month as shown in Figure 1 and Supplementary Tables (Table 2).

### Effect of gender-genotypes interaction of recipients on Tacrolimus pharmacokinetics parameters

Recipients were grouped according to their gender (male vs female) then categorized into two subgroups according to the presence of at least one ancestral allele versus the homozygous variant genotype (-592; CC and CA vs AA), (-819; CC and CT vs TT) and (-1082; AA and AG vs GG). All mentioned parameters did not differ significantly among*IL-10 *(-592, rs1800872, A/C); *IL-10* (–819, rs1800871, T/C) during the first six months post-transplantation (as shown in *Supplementary Tables*, Table 5).

**Table 5:** Haplotype Distribution of IL-10 –592, –819 and –1082 among renal transplantation recipients one month post transplantation N: number of recipients. C (first): the ancestral allele of IL-10 –592. C (middle): the ancestral allele of IL-10 –819. A (last) the ancestral allele of IL-10 –1082. A (first): the variant allele of IL-10 –592. T (middle): the variant allele of IL-10 –819. G (last): the variant allele of IL-10–1082

Haplotype	Total (%)	<= median<br>N=27 (%)	>median<br>N=23 (%)	P
ATA	31.6	7(27.3)	8(36.7)	0.99
CCG	29.9	6(24.8)	8(35.9)	0.99
CCA	27.6	11(41.1)	3(11.7)	0.04
ACA	3.6	1(1.8)	1(5.7)	0.94
ACG	3.0	0(1.0)	1(5.4)	
CTA	2.1	1(2.0)	1(2.3	0.94
CTG	2.1	1(2.0	1(2.3)	0.94

**Figure 2:**
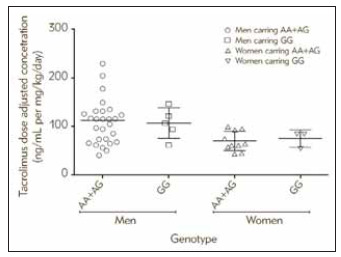
Effect of gender-genotype interaction at *IL-10* (–1082, rs1800896, A/G) on Tacrolimus dose adjusted concentration (ng/mL per mg/kg/day) of Jordanian kidney transplant recipients in the first month post transplantation Y-axis is dose adjusted concentration (ng/mL per mg/kg/day),<br>X-axis is the genotypes at *IL-10* –1082. AA: Homozygote ancestral genotype, GG: Homozygous variant genotype and AG: Homozygous variant genotype

However, we found that patients carrying GG genotype at*IL-10* (–1082, rs1800896, A/G) versus patients carrying at least one A allele (AA or AG) show differences. Men carrying at least one A allele had significantly lower Tacrolimus adjusted concentration than men carrying GG genotype in the first month post-transplantation. This reduction in DAC, however, disappeared after the first month [88.2±32.1 vs. 117.5±22.5 ng/mL per mg/kg/day, p=0.04]. On the other hand, in women, non of mentioned parameters differed significantly between different *IL-10 *-1082 A>G genotype groups during the first six months post-transplantation as shown in Figure 2 and Supplementary Tables (Table 4).

## Discussion

This study examined the contribution of IL-10 (-592, rs1800872, A/C); IL-10 (–819, rs1800871, T/C) and IL-10 (–1082, rs1800896, A/G)polymorphisms in Jordanian renal transplant recipients to Tacrolimuspharmacokinetics parameters within the first six months post-transplantation. The clinical use of Tacrolimus is complicated by its narrow therapeutic range and highly variable pharmacokinetics among individuals. Some patients do not reach target concentrations using the recommended initial doses of Tacrolimus, and therefore, have an increased risk of inadequate immunosuppression and subsequent acute rejection during the early period following organ transplantation [20]. The association of the IL-10 gene SNPs with Tacrolimus dose requirements has been recognized as a genetic base for the observed inter-individual differences in pharmacokinetics [21].

Our current study aimed to determine whether the genotype of IL-10 could explain variability in pharmacokinetic parameters of Tacrolimus in kidney recipients during the proposed period. We hypothesized that the recipient's polymorphisms of IL-10 are associated with changes in Tacrolimuspharmacokinetics parameters during the early period post-transplantation.

In our current study, IL-10 alleles frequencies were found to be as follows; the A allele: 65% and the G allele: 35%. this is consistent with data from previously published research on such frequencies among Caucasians (A allele: 58.5–65.6%, G allele: 34–41.5%) [22] [23].

Turner and Williams [15] found that following stimulation, IL-10 production was measured by ELISA showed that the GG genotype -1082 is significantly higher compared to the AA and AG genotypes. This correlation was independent of the polymorphisms at positions -819 and -592. Later, studies found that the G allele at position -1082 is the most important genetic factor in the regulation of constitutive IL-10 mRNA level, and is associated with a greater serum concentration [16] [17]. Furthermore, an important relationship was noted between IL-10 and cytochrome P450 activity through an *in vivo* study that showed IL-10 to significantly decrease CYP3A activity (P ≤ 0.02) [18]. Interestingly, a previous study conducted among Jordanian kidney transplant recipients revealed a correlation between genetic variations in both CYP3A4 and CYP3A5 enzymes and tacrolimus blood levels among renal transplant recipients [13].

In a previous study of liver transplant recipients, significantly higher Tacrolimus dose-adjusted concentrations were measured in patients carrying -1082 AA versus those carrying GG and GA during an intermediate value within the first three weeks after transplantation [21]. On the other hand, a Chinese study demonstrated the impact of IL-10 gene polymorphism on Tacrolimus dosage requirement in 53 liver transplant recipients and found no statistically significant differences in Tacrolimus dose-adjusted concentration among recipients. The same study revealed a significantly higher Tacrolimus dose-adjusted concentration in recipients with donors with the -1082 AA genotype than those whose donors with IL-10 -1082 GA genotype [24].

In a later study including 240 renal transplant recipients, IL-10 (-1082) variants did not show a significant relationship between Tacrolimus metabolism and -1082 genotypes within the first four weeks following transplantation [25]. The current study did not find a significant relationship between studied IL-10 SNPs among kidney transplant recipients and Tacrolimus pharmacokinetics parameters. Remarkably, gender analysis revealed that males carrying at least one A allele at IL-10 (-1082) had significantly lower Tacrolimus dose-adjusted concentration than males carrying GG genotype in the first-month post-transplantation. We divided the patients according to their gender due to the differences in liver and renal function between males and females [26].

Our results can be explained by hypothesizing that the -1082 GG allele is associated with increased IL-10 production [15] [16] [17], which leads to decreased CYP3A catalytic activity [18]. Hence, a lower tacrolimus dose is required to reach a significantly higher Tacrolimus dose-adjusted concentration. This is evident during the early phase after transplantation.

Multiple studies have demonstrated linkage disequilibrium between the polymorphism at position -1082 in the IL-10 promoter area and other SNPs in the same area including SNPs at positions -819 and -592, suggesting that the functional effects may be haplotype-dependent [27] [25] [21].

The current study shows that Tacrolimus adjusted concentration is sex-genotype-dependent in Jordanian kidney transplant recipients during the first-month post-transplantation at IL-10 -1082 A>G. This effect was observed in the first-month post-transplantation in male patients carrying at least one A allele who showed significantly lower DAC than male patients carrying the GG genotype. This reduction in DAC disappeared after the first month. On the other hand, non of the mentioned parameters differed significantly between different IL-10 -1082 A>G genotype groups during the first six months post-transplant in the female patients.

### Limitations

The number of studied patients was small due to the long follow-up period of 6 months per patient. As well there were cases where data was missing due to the difficulty in interviewing patients, or loss of contact with patients. Because of the small sample size, we couldn't detect rare mutations and their frequency impact on tacrolimus pharmacokinetic parameters. However, it should be noted that our sample size is similar to other previously published studies that were close to (240) or even smaller (53) than the present study [21] [24] [25].

## Dodatak

### Acknowledgment

This research was supported by unconditional support by Yarmouk University (15/2017) and the University of Jordan/Deanship of Academic Research (469/2017). We gratefully thank the local research ethics committee of Royal Medical Services and all the patients who agreed to participate.

### Conflict of interest statement

All the authors declare that they have no conflict of interest in this work.

### List of abbreviations

ESRD, End-stage renal disease;<br>SNPs, Single-nucleotide polymorphisms;<br>SD, Standard deviation;<br>BSA, Body surface area;<br>DAC, Dose-adjusted concentration
